# Downregulated TNF-α Levels after Cryo-Thermal Therapy Drive Tregs Fragility to Promote Long-Term Antitumor Immunity

**DOI:** 10.3390/ijms22189951

**Published:** 2021-09-15

**Authors:** Yue Lou, Junjun Wang, Peng Peng, Shicheng Wang, Ping Liu, Lisa X. Xu

**Affiliations:** Med-X Research Institute, School of Biomedical Engineering, Shanghai Jiao Tong University, Shanghai 200030, China; louyue4@163.com (Y.L.); sjtuwangjunjun@sjtu.edu.cn (J.W.); 112358answer@sjtu.edu.cn (P.P.); shichengwang@sjtu.edu.cn (S.W.)

**Keywords:** cryo-thermal therapy, Treg fragility, TNF-α

## Abstract

Immunotherapy has emerged as a therapeutic pillar in tumor treatment, but only a minority of patients get benefit. Overcoming the limitations of immunosuppressive environment is effective for immunotherapy. Moreover, host T cell activation and longevity within tumor are required for the long-term efficacy. In our previous study, a novel cryo-thermal therapy was developed to improve long-term survival in B16F10 melanoma and s.q. 4T1 breast cancer mouse models. We determined that cryo-thermal therapy induced Th1-dominant CD4^+^ T cell differentiation and the downregulation of Tregs in B16F10 model, contributing to tumor-specific and long-lasting immune protection. However, whether cryo-thermal therapy can affect the differentiation and function of T cells in a s.q. 4T1 model remains unknown. In this study, we also found that cryo-thermal therapy induced Th1-dominant differentiation of CD4^+^ T cells and the downregulation of effector Tregs. In particular, cryo-thermal therapy drove the fragility of Tregs and impaired their function. Furthermore, we discovered the downregulated level of serum tumor necrosis factor-α at the late stage after cryo-thermal therapy which played an important role in driving Treg fragility. Our findings revealed that cryo-thermal therapy could reprogram the suppressive environment and induce strong and durable antitumor immunity, which facilitate the development of combination strategies in immunotherapy.

## 1. Introduction

Cancer therapy has long depended on strategies that directly attack tumor cells. Recently, cancer treatment has been revolutionized by the development of various immunotherapeutics. Cancer immunotherapy harnesses the patient’s immune system to fight cancer and now provides a new treatment option in the therapeutic paradigm of surgery, radiotherapy, and chemotherapy. However, the therapeutic efficacy of immunotherapy is challenged by the low response rate and acquired resistance caused by tumor genetic instability [1]. One of the key factors that promote resistance is the suppressive tumor environment [2]. Moreover, increasing evidence shows that the activation of endogenous tumor-specific T cell responses is essential to obtain the clinical benefits of tumor immunotherapy, such as immune checkpoint inhibition [3], chimeric antigen receptor T-cell immunotherapy (CAR-T) [4], and DC vaccines [5]. Thus, ongoing immunotherapy studies are needed to develop strategies to overcome various immune tolerances and to promote the activation of host T cells.

Cytotoxic CD8^+^ T cells are known to provide effective antigen-specific immunity against tumors, which can directly kill tumor cells through the release of Granzyme and perforin or the activation of the Fas ligand pathway [6]. CD4^+^ T cells, as a key component of the immune system, are central in orchestrating adaptive immune responses [7]. Since CD4^+^ T cells can independently kill tumor cells and promote the immune response through the regulation of other innate and adaptive immune cells, they were recently considered to play a more important role in the antitumor response [8]. CD4^+^ T cells display a large degree of plasticity to differentiate into CD4 CTL, Th1, Th2, Th17, Tregs, and Tfh subsets, but the polarization of CD4^+^ T cells determines the immune balance of the antitumor response. Among CD4^+^ T subsets, Th1 is the main subpopulation that facilitates tumor regression and a cure [9]. Th1 cells expressing interferin-γ (IFN-γ) are capable of recognizing and killing major histocompatibility complex (MHC)-II-expressing tumors directly, as well as promoting the recruitment and infiltration of immune cells, stimulating antigen presentation and costimulatory molecule expression of antigen-presenting cells (APCs), and they encourage the cytotoxicity of CD8^+^ T and nature killer (NK) cells [7,10]. In contrast, Tregs are continuously involved in tumor progression through the upregulated expression of immune checkpoint molecules and suppressive cytokines, which inhibit antigen presentation of dendritic cells (DCs) and induce exhaustion of other T cells [11]. Abundant infiltration of Tregs in the tumor environment often correlates with poor efficacy [12]. Thus, a balance in favor of Th1 differentiation is one of the most important issues for obtaining the maximum effective antitumor response [13]. The depletion of Tregs or the specific blockade of suppressive molecules on Tregs can attenuate suppression and stimulate the function of effector T cells [14].

In our previous studies, we developed a novel tumor cryo-thermal therapy through alternative liquid nitrogen cooling and radiofrequency (RF) heating of tumor tissue and used it to treat s.q. 4T1 murine breast cancer and B16F10 melanoma models. In B16F10 melanoma models, the survival rate of mice after cryo-thermal therapy was more than 80%; moreover, cryo-thermal treated mice could resist tumor rechallenge [15,16]. In the s.q. 4T1 murine breast cancer model, almost 50% of mice had long-term survival after cryo-thermal therapy [17]. Through in-depth immunological mechanistic studies we found that cryo-thermal therapy induced Th1-dominant CD4^+^ T cell differentiation and downregulated the proportion of Tregs in B16F10 melanoma models, which facilitated tumor-specific and robust immune protection [15,16]. In the s.q. 4T1 murine breast cancer model, we revealed that cryo-thermal therapy could induce an acute immune response by triggering the expression of damage-associated molecular patterns (DAMPs) and interleukin (IL)-6 [18,19]. However, whether cryo-thermal therapy affects the differentiation and function of T cells in a s.q. 4T1 murine breast cancer model remains to be explored.

In this study, we investigated the therapeutic efficacy of cryo-thermal therapy in a s.q. 4T1 murine breast cancer model and on the differentiation of T cells. We found that cryo-thermal therapy could prevent tumor metastasis and increase long-term survival in a s.q. 4T1 murine breast cancer model. Moreover, cryo-thermal therapy could induce Th1-dominant polarization of CD4^+^ T cells and decrease the frequency of Tregs. In particular, cryo-thermal therapy could drive the fragility of Tregs and impair the suppressive function of Tregs. Furthermore, we verified that the decreased level of serum tumor necrosis factor (TNF)-α at the late stage after cryo-thermal therapy played an important role in driving Tregs fragility. This study revealed that cryo-thermal therapy could reprogram the tumor suppressive environment and elicit strong and durable systemic T cell-mediated antitumor immunity, which would help develop novel antitumor strategies for synergistic combinations of immunotherapies.

## 2. Results

### 2.1. Cryo-Thermal Therapy Promoted Long-Term Antitumor Immune Protection of Distant Tumor Metastasis

**Figure 1:**
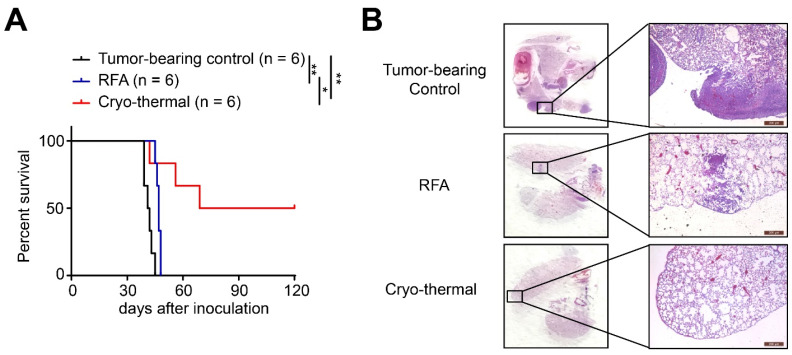
Cryo-thermal therapy induced long-term antitumor protection in 4T1-bearing mice. (**A**) Kaplan–Meier survival curve of tumor-bearing control, traditional RFA or cryo-thermal therapy treated mice. Approximately 4 × 10^5^ 4T1 cells were injected subcutaneously into the right flank of female Balb/c mouse. Then, 18 days later, mice were randomly allocated into three groups, and treated by RFA or cryo-thermal therapy, and untreated mice were as control. Kaplan–Meier survival curve was compared using log-rank tests. * *p* < 0.05, ** *p* < 0.01. *n* = 6 for each group. (**B**) H&E staining of the lung of tumor-bearing control (39 days after inoculation), RFA or cryo-thermal treated group (21 days after treatment).

In our previous studies, novel tumor cryo-thermal therapy was performed in s.q. 4T1 murine breast cancer and B16F10 murine melanoma models, and cryo-thermal therapy significantly improved the survival rate in both mouse models [17,18,20]. To confirm the therapeutic effect of cryo-thermal therapy, the survival experiment in cryo-thermal treated 4T1 tumor-bearing mice was repeated. As shown in Figure 1A, all of the tumor-bearing mice died 7 weeks after tumor inoculation, while radiofrequency ablation (RFA) treatment slightly prolonged the survival time of mice (the median survival time was approximately 47 days), but all mice died due to tumor metastasis. These data indicated that conventional RFA only prolonged the survival time of mice, but all mice succumbed to their tumors from tumor progression. However, cryo-thermal therapy significantly improved the survival rates and prolonged the survival time of mice. Further H&E staining of the lungs from tumor-bearing mice, RFA- and cryo-thermal treated mice showed that cryo-thermal therapy could efficiently inhibit lung metastasis compared to mice treated with RFA. The results suggested that local cryo-thermal therapy induced systemic antitumor immunity and conferred long-term protection against tumor metastasis.

### 2.2. Cryo-Thermal Therapy Promoted CD4^+^ T Cell Proportion and Th1 Differentiation in the Late Stage after Treatment

**Figure 2:**
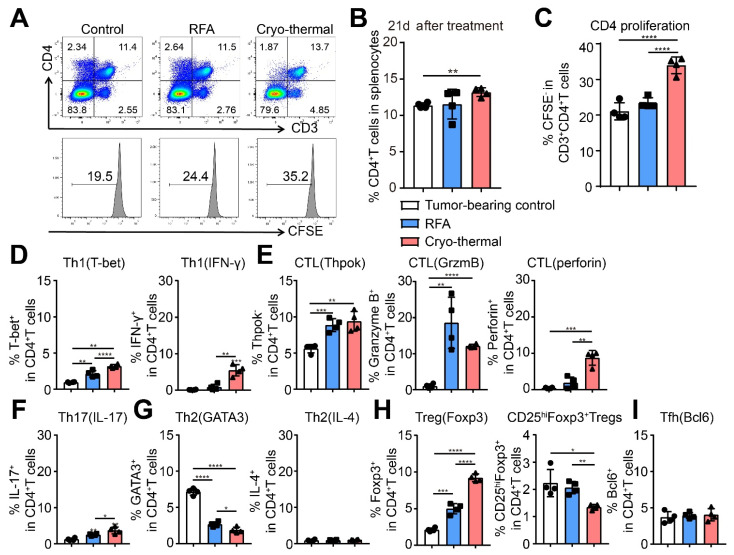
Cryo-thermal therapy promoted CD4^+^ T cell-mediated antitumor immune response. (**A**–**C**) The percentages of CD4^+^ T cells of splenic lymphocytes (**B**) and the proliferation (**C**) of CD4^+^ T cells were detected by flow cytometry on day 21 after cryo-thermal therapy or RFA treatment. For CD4^+^ T cells proliferation assay, splenic CD4^+^ T cells were isolated from tumor-bearing control (39 days after inoculation), RFA or cryo-thermal (21 days after treatment) treated mice by using microbeads. The CD4^+^ T cells were labeled with CFSE and then cultured with αCD3 (1 ng/mL) for 24 h. The proliferation of CD4^+^ T cells (CFSE^−^) was detected by flow cytometry. (**D**–**I**) The percentages of CD4 Th1 (**D**), CTL (**E**), Th17 (**F**), Th2 (**G**), Tregs (**H**), and Tfh (**I**) subsets in spleen were detected by flow cytometry. * *p* < 0.05, ** *p* < 0.01, *** *p* < 0.001, **** *p* < 0.0001. *n* = 4 for each group.

The long-term antitumor response is considered to be mediated by Th1-type differentiation of CD4^+^ T cells [9]. In our previous study, cryo-thermal therapy induced strong and long-lasting Th1 antitumor immune memory in B16F10 melanoma models [15]. To investigate whether CD4^+^ T cells also played a major role in the s.q. 4T1 murine breast cancer model after cryo-thermal therapy, the proportion of splenic CD4^+^ T cells from tumor-bearing, RFA- or cryo-thermal treated mice was detected by using flow cytometry. On Day 21 after treatment, the percentage of splenic CD4^+^ T cells from cryo-thermal treated mice was much higher than that in the RFA-treated group (Figure 2A,B). Cell proliferation assays in vitro was detected by CFSE. In the process of CFSE staining, the three different samples were analyzed separately, resulting in slightly different mean fluorescence intensity of the unproliferated peak values in the three groups. Thus, Flowjo was used to analyze the gating strategy of proliferated cells. The peak of unproliferated cells and gating of proliferated cells were determined by the result of Flowjo. Results also demonstrated that splenic CD4^+^ T cells from cryo-thermal treated mice had a higher proliferation potential than those from control mice and RFA-treated mice (Figure 2A,C). These data indicated that cryo-thermal therapy could amplify CD4^+^ T cells at a high level.

Then, we investigated the subsets of CD4^+^ T cells on day 21 after treatment. The results showed that the percentage of Th1 cells (CD4^+^T-bet^+^) was upregulated after RFA and cryo-thermal therapy compared to the tumor-bearing control, but the level of Th1 cells after cryo-thermal therapy was much higher than that after RFA treatment. Meanwhile, the expression of the Th1 cytokine IFN-γ in CD4^+^ T cells after cryo-thermal therapy was dramatically upregulated compared to that in tumor-bearing control and RFA-treated mice (Figure 2D). At the same time, the percentage of CD4 CTLs (CD4^+^Thpok^−^) and the expression of the CTL cytokine Granzyme B in CD4^+^ T cells were notably increased after RFA and cryo-thermal therapy compared to that in tumor-bearing mice. However, after cryo-thermal therapy, the expression level of the CTL cytokine perforin in CD4^+^ T cells was significantly upregulated compared with that in the tumor-bearing control and RFA groups (Figure 2E). Furthermore, the percentage of the Th17 subset (CD4^+^IL-17^+^) was increased after RFA and cryo-thermal therapy, but the level of Th17 cells in cryo-thermal treated mice was higher than that in RFA-treated mice (Figure 2F). The proportion of the Th2 subset (CD4^+^GATA3^+^) was decreased after RFA and cryo-thermal therapy compared to that of tumor-bearing mice, and the level of Th2 cells in the cryo-thermal treated group was much lower than that in the RFA group (Figure 2G). The expression of the Th2 cytokine IL-4 in CD4^+^ T cells did not change among the three groups (Figure 2G). Interestingly, the percentage of Tregs (CD4^+^Foxp3^+^) was upregulated in the RFA and cryo-thermal treated groups compared with the tumor-bearing mice, and the proportion of Tregs in the cryo-thermal treated group was much higher than that in the RFA group. However, the percentage of effector Treg subsets (CD4^+^CD25^hi^Foxp3^+^) after cryo-thermal therapy was significantly downregulated compared to tumor-bearing control and RFA-treated mice (Figure 2H). The percentage of the Tfh subset had no obvious change among the three groups (Figure 2I). These results indicated that cryo-thermal therapy could induce Th1 polarization of CD4^+^ T cells and increase the cytotoxicity of CD4^+^ T cells while downregulating the differentiation of Th2 and effector Treg subsets.

The proportion and cytotoxicity of CD8^+^ T cells after treatment were also investigated. However, in comparison with tumor-bearing mice, the percentages of CD8^+^ T cells were increased after RFA and cryo-thermal therapy, but the proliferation of CD8^+^ T cells was suppressed in the two groups (Figure S1). Furthermore, although the expression of the cytokines IFN-γ and perforin in CD8^+^ T cells was upregulated after RFA and cryo-thermal therapy compared to tumor-bearing mice, the expression levels of IFN-γ and Granzyme B in CD8^+^ T cells after cryo-thermal therapy were much lower than those in the RFA group (Figure S1). These results suggested that CD8^+^ T cells do not play a major role in antitumor immunity after cryo-thermal therapy.

### 2.3. Cryo-Thermal Therapy Increased the Fragility of Tregs and Downregulated Their Suppressive Function

**Figure 3:**
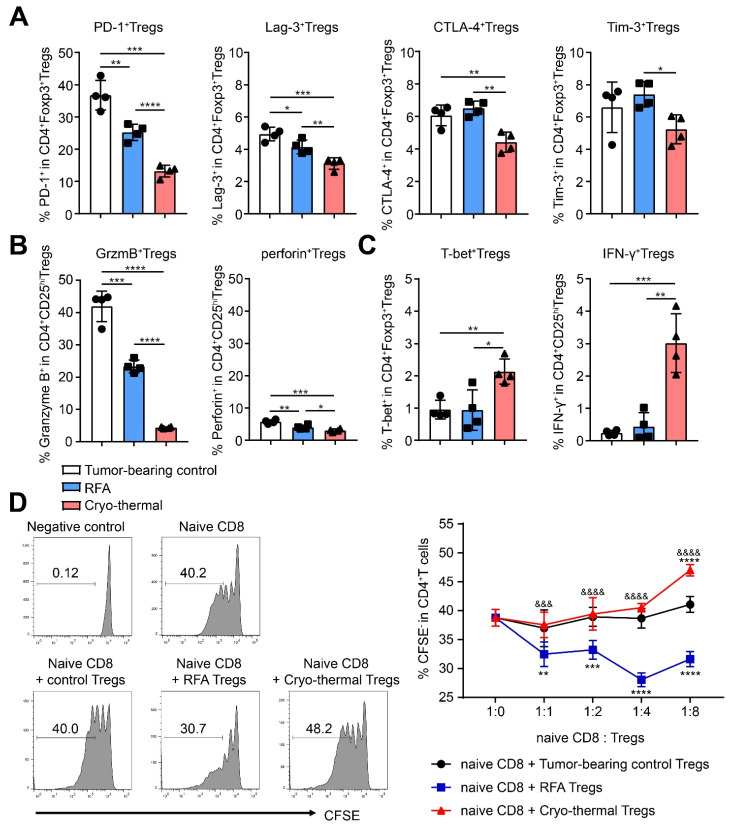
Cryo-thermal therapy drove the fragile phenotype of Tregs and down-regulated its suppressive function. (**A**–**D**) The expression level of inhibitory checkpoint molecules (**A**), suppressive cytokines Granzyme B and perforin (**B**), and T-bet and IFN-γ expression (**C**) on splenic Tregs from tumor-bearing control (39 days after inoculation), RFA or cryo-thermal therapy (21 days after treatment) were detected by flow cytometry. (**D**) Splenic Tregs from tumor-bearing control (39 days after inoculation), RFA or cryo-thermal therapy (21 days after treatment) and splenic CD8^+^ T cells from naïve mice were isolated through microbeads. CD8^+^ T cells were labeled with CFSE and then co-cultured with Tregs in different E/T ratio. αCD3 (1 ng/mL) was used to stimulated the proliferation of CD8^+^ T cells. After 72 h, the proliferated CD8^+^ T cells (CFSE-) were tested by flow cytometry. * *p* < 0.05, ** *p* < 0.01, *** *p* < 0.001, **** *p* < 0.0001 compared with the E/T ratio of 0:1. ^&&&^ *p* < 0.001, ^&&&&^ *p* < 0.0001 compared with the same E/T ratio of group RFA. *N* = 4 for each group.

Tregs have been described to maintain immune tolerance through the increased expression of inhibitory checkpoint molecules and suppressive cytokines [21]. In particular, the expression of Granzyme B and perforin in Tregs could induce the death of NK and CD8^+^ T cells and suppress tumor clearance in vivo [22]. Thus, the expression of inhibitory checkpoints and suppressive cytokines in Tregs was determined. Compared to tumor-bearing mice, the expression of programmed cell death protein-1 (PD-1) and lymphocyte-activation gene 3 (Lag-3) on Tregs was clearly downregulated after RFA. After cryo-thermal therapy, the expression of PD-1, cytotoxic T-lymphocyte-associated antigen 4 (CTLA-4), Lag-3 and T cell immunoglobulin and mucin domain-containing-3 (Tim-3) on Tregs was markedly decreased and was much lower than that in RFA-treated mice (Figure 3A). At the same time, the expression of Granzyme B and perforin in Tregs was decreased after RFA and cryo-thermal therapy compared to tumor-bearing mice; however, after cryo-thermal therapy, the levels of Granzyme B and perforin in Tregs were much lower than those in the RFA group (Figure 3B). These data indicated that cryo-thermal therapy impaired the inhibitory signature of Tregs.

Treg fragility is defined as the retention of Foxp3 expression with loss of suppressive function. Fragile Tregs produce IFN-γ and upregulate the IFN-γ receptor, as well as the transcription factor T-bet. Fragile Tregs reduce the expression of suppressive molecules and are functionally less suppressive [23]. The development of combinatorial immunotherapies that maximize Treg fragility may be the most effective ways to improve patient response to immunotherapy [24]. Considering that the proportion of total Tregs (Foxp3^+^) was upregulated but the percentage of effector Tregs was downregulated after cryo-thermal therapy (as shown in Figure 2H) and that the expression of suppressive molecules in Tregs was downregulated, we hypothesized that cryo-thermal therapy could drive the fragility of Tregs. As shown in Figure 3D, the expression of T-bet and IFN-γ in Tregs were obviously increased after cryo-thermal therapy compared with that in tumor-bearing mice and RFA treatment. All the above results suggested that cryo-thermal therapy drove the fragility of Tregs and attenuated the suppressive activity of Tregs on day 21 after treatment, which boosted antitumor immunity.

Tregs can directly inhibit the proliferation of effector T cells [25]. To investigate whether cryo-thermal therapy impaired the suppressive function of Tregs on Day 21 after treatment, splenic Tregs from tumor-bearing control, RFA or cryo-thermal treated mice were isolated and cocultured with splenic CD8^+^ T cells from age-matched mice. The proliferation of CD8^+^ T cells was analyzed by CSFE staining. Splenic Tregs from RFA-treated mice could significantly suppress the proliferation of CD8^+^ T cells compared with those from tumor-bearing control mice; however, splenic Tregs from cryo-thermal treated mice did not have a suppressive function at a low E/T ratio but instead promoted the proliferation of CD8^+^ T cells at a high E/T ratio. These results further demonstrated that cryo-thermal therapy impaired the suppressive function of Tregs on day 21 after treatment.

### 2.4. Cryo-Thermal Therapy Downregulated the Level of Serum TNF-α

**Figure 4:**
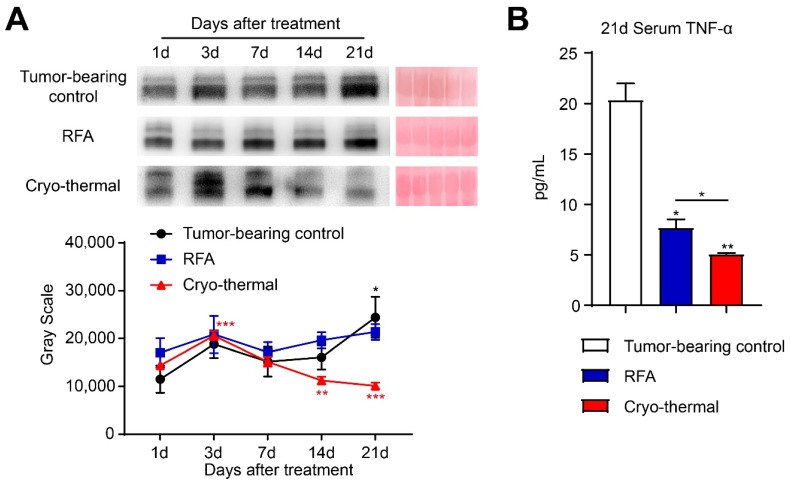
The level of serum TNF-α was decreased after cryo-thermal therapy. (**A**) The level of serum TNF-α from tumor-bearing control, RFA or cryo-thermal treated mice on different time points was detected by western blot (up), and the gray statistics of three independent experiments (down). (**B**) The level of serum TNF-α from tumor-bearing control, RFA or cryo-thermal treated mice on day 21 after treatment was detected by using ELISA. The gray scale was analyzed by image J. * *p* < 0.05, ** *p* < 0.01, *** *p* < 0.001. *n* = 3 for each group.

TNF-α is one of the most important cytokines that contributes to chronic inflammation and promotes the accumulation of Tregs in the tumor environment [26]. Moreover, TNF-α can increase the capacity of Tregs to inhibit proliferation and IFN-γ production by effector T cells [27]. To investigate the mechanism of Treg fragility and the downregulated suppressive function of Tregs after cryo-thermal therapy, the level of serum TNF-α in tumor-bearing control mice, RFA-treated mice or cryo-thermal treated mice was measured at different time points (1 d, 3 d, 7 d, 14 d, and 21 d after treatment) by western blotting and further verified by ELISA on day 21 after treatment. The level of serum TNF-α was increased with tumor growth in tumor-bearing control mice, but in the RFA-treated group, the level of TNF-α had no obvious change after treatment (Figure 4A). Interestingly, after cryo-thermal therapy, the level of serum TNF-α was upregulated on day 3 and then declined gradually over time (Figure 4A). Then, the level of serum TNF-α in these three groups was further verified by ELISA on day 21 after treatment. On day 21 after cryo-thermal therapy, the level of serum TNF-α was significantly lower than that in tumor-bearing control and RFA-treated mice (Figure 4B). Considering that the decreased serum TNF-α and the downregulated suppressive activity of Tregs occurred at the same time point (on Day 21 after cryo-thermal therapy), we hypothesized that the decreased level of TNF-α after cryo-thermal therapy drove Tregs fragility.

### 2.5. TNF-α Supplementation after Cryo-Thermal Therapy Prevented Treg Fragility and Promoted the Suppressive Function of Tregs

**Figure 5:**
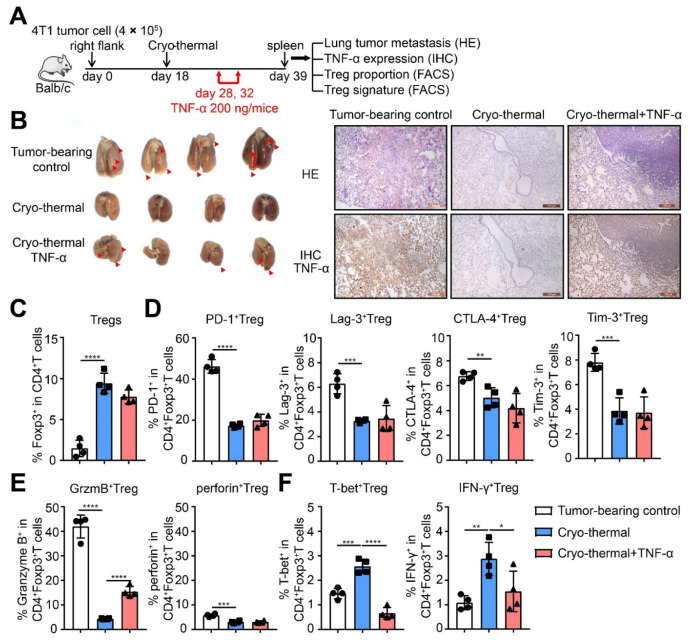
In vivo TNF-α supplement after cryo-thermal therapy damaged Tregs fragility and promoted tumor metastasis of mice. (**A**) Schematic of experimental design. For TNF-α supplement in vivo, 200 ng recombinant TNF-α protein was injected intraperitoneally in 100 μL PBS on day 10 and 14 after cryo-thermal therapy. (**B**) Photographic images of lungs (left), HE staining (upper-right) and IHC staining of TNF-α (brown, lower right) from tumor-bearing control (39 days after inoculation), cryo-thermal treated or TNF-α supplied after cryo-thermal therapy mice (21 days after treatment). (**C**–**E**) The proportion of Tregs (**C**), the expression of inhibitory checkpoints (**D**), the expression of suppressive cytokines (**E**), and the expression of T-bet and IFN-γ (**F**) of Tregs were detected on day 21 after single cryo-thermal, or combined with TNF-α supplement by flow cytometry. *n* = 4 for each group. * *p* < 0.05, ** *p* < 0.01, *** *p* < 0.001, **** *p* < 0.0001.

To reveal the role of TNF-α in the differentiation and suppressive function of Tregs and considering that the level of serum TNF-α was much lower after cryo-thermal therapy, 200 ng recombinant TNF-α was injected intraperitoneally on days 10 and 14 after cryo-thermal therapy. As shown in Figure 5B, cryo-thermal therapy with TNF-α supplementation significantly increased lung metastasis compared to cryo-thermal therapy alone. At the same time, the level of pulmonary TNF-α was detected by immunohistochemistry assays. The level of TNF-α in the lungs of tumor-bearing mice and mice treated with cryo-thermal therapy with TNF-α supplementation was much higher than that after cryo-thermal therapy alone. All of the above results indicated that the decreased level of TNF-α after cryo-thermal therapy inhibited tumor metastasis (Figure 5B).

**Figure 6:**
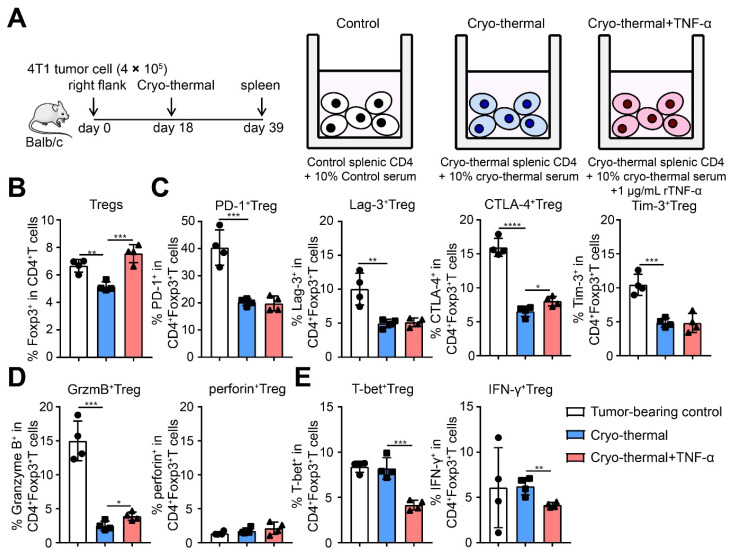
In vitro TNF-α supplement after cryo-thermal therapy damaged Tregs fragility and promoted tumor metastasis of mice. (**A**) Schematic of experimental design. Splenic CD4^+^ T cells were separated from tumor-bearing control (39 days after inoculation) or cryo-thermal treated mice (21 days after treatment) and cultured in vitro for 24 h. The serum from tumor-bearing or cryo-thermal treated mice was added in corresponding group. Recombinant TNF-α protein was added in the concentration of 1 μg/mL. The proportion of Tregs (**B**), the expression of inhibitory checkpoints (**C**), the expression of suppressive cytokines (**D**), and the expression of T-bet, IFN-γ of Tregs (**E**) were detected by flow cytometry. *n* = 4 for each group. * *p* < 0.05, ** *p* < 0.01, *** *p* < 0.001, **** *p* < 0.0001.

Next, we determined the proportion and suppressive signature of Tregs after cryo-thermal therapy with TNF-α supplementation. TNF-α supplementation after cryo-thermal therapy had less effect on the proportion of Tregs and the expression of PD-1, Lag-3, CTLA-4, and Tim-3 on Tregs than cryo-thermal therapy alone (Figure 5C,D). However, the expression of Granzyme B on Tregs was significantly upregulated, and the expression of perforin had no change after cryo-thermal therapy with TNF-α supplementation (Figure 5E). Interestingly, TNF-α supplementation after cryo-thermal therapy downregulated the expression of T-bet and IFN-γ on Tregs compared to cryo-thermal therapy alone (Figure 5F). To further confirm the effects of TNF-α on the function of Tregs after cryo-thermal therapy, splenic CD4^+^ T cells from tumor-bearing control (39 days after inoculation) or cryo-thermal treated mice (21 days after treatment) were isolated and cultured in vitro. The serum of tumor-bearing or treated mice was added to the corresponding group to mimic the in vivo situation. Recombinant TNF-α was administered to splenic CD4^+^ T cells from cryo-thermal treated mice (1 μg/mL), which were cultured for 24 h. Then, we determined the proportion and suppressive signature of Tregs after cryo-thermal therapy with TNF-α supplementation in vitro. The proportion of Tregs in splenic CD4^+^ T cells from cryo-thermal treated mice was decreased compared with that in the tumor-bearing control, but TNF-α supplementation after cryo-thermal therapy increased the proportion of Tregs compared to cryo-thermal therapy alone (Figure 6B). These results suggested that TNF-α could promote the accumulation of Tregs. At the same time, the expression of PD-1, Lag-3, CTLA-4, Tim-3, and Granzyme B on Tregs from cryo-thermal treated mice was decreased, and the expression of perforin, T-bet and IFN-γ in Tregs was not different from that in Tregs from tumor-bearing control mice (Figure 6C–E). However, TNF-α supplementation after cryo-thermal therapy obviously increased the expression of CTLA-4 and Granzyme B on Tregs and decreased the expression of T-bet and IFN-γ, although the levels of PD-1, Lag-3, Tim-3, and perforin on Tregs showed no obvious changes compared to cryo-thermal therapy alone (Figure 6C–E). Together, these in vivo and vitro results indicated that the decreased level of TNF-α after cryo-thermal therapy clearly drove the fragility of Tregs and impaired the suppressive function of Tregs.

### 2.6. TNF-α Neutralization after RFA Treatment Inhibited Tregs Differentiation and Tumor Metastasis

**Figure 7:**
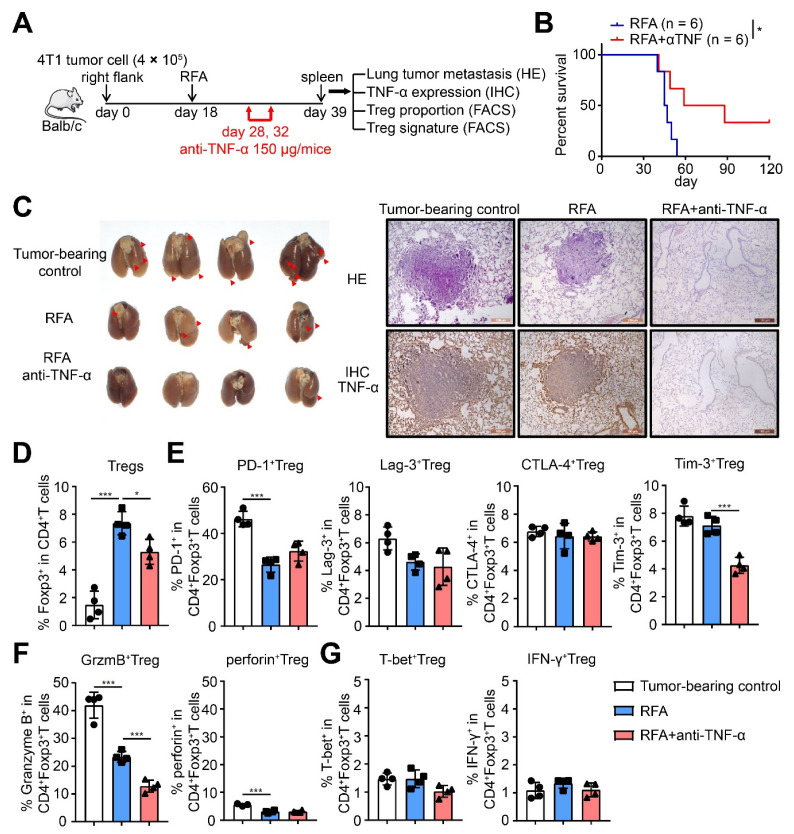
TNF-α neutralization after RFA treatment inhibited Treg function and suppressed tumor metastasis, as well as promoted the long-term survival of mice. (**A**) Schematic of experimental design. For TNF-α neutralization in vivo, 150 μg antibody was injected intraperitoneally in 100 μL PBS on day 10 and 14 after RFA treatment. (**B**) Kaplan–Meier survival curve of single RFA therapy or combination with TNF-α neutralization. *n* = 6 for each group. (**C**) Photographic images of lungs (left), HE staining (upper-right) and IHC staining of TNF-α (brown, lower right) from tumor-bearing control (39 days after inoculation), RFA or cryo-thermal treated (21 days after treatment) mice. (**D**–**G**) The proportion of Tregs (**D**), the expression of inhibitory checkpoints (**E**), the expression of suppressive cytokines (**F**) and the expression of T-bet and IFN-γ (**G**) of Tregs were detected on day 21 after single RFA, or combined with TNF-α neutralization by flow cytometry. *n* = 4 for each group. * *p* < 0.05, *** *p* < 0.001.

To further elucidate the role of TNF-α in the differentiation and suppressive function of Tregs and considering that the level of serum TNF-α after RFA treatment was much higher than that after cryo-thermal therapy, TNF-α was neutralized with an antibody on days 10 and 14 after RFA treatment. As shown in Figure 7B, TNF-α neutralization after RFA treatment significantly increased the long-term survival of 4T1-bearing mice compared to RFA treatment alone. On day 21, TNF-α neutralization after RFA treatment notably reduced lung metastasis compared with that in tumor-bearing mice and after RFA treatment alone (Figure 7C). At the same time, as shown in Figure 6C, immunohistochemistry assays showed that the level of pulmonary TNF-α after RFA with TNF-α neutralization was much lower than that from tumor-bearing mice and after RFA treatment alone (Figure 7C), which indicated that a higher level of TNF-α after RFA treatment promoted tumor metastasis.

After RFA treatment with TNF-α neutralization, the percentage of Tregs was significantly decreased (Figure 7D); at the same time, the expression of Tim-3 and Granzyme B on Tregs was notably downregulated compared to RFA treatment alone, although the expression of PD-1, CTLA-4, and Lag-3 on Tregs had no obvious changes (Figure 7E,F). However, the expression of T-bet and IFN-γ in Tregs showed no significant change (Figure 7G). An in vitro assay was performed to evaluate the effects of TNF-α on regulating the function of Tregs after RFA treatment. Splenic CD4^+^ T cells from tumor-bearing control (39 days after inoculation) or RFA-treated mice (21 days after treatment) were isolated and cultured in vitro. The serum of tumor-bearing or treated mice was administered to the corresponding group to mimic the in vivo situation. Anti-TNF-α antibody was used to neutralize TNF-α release in splenic CD4^+^ T cells from RFA-treated mice (10 μg/mL), which were cultured for 24 h.

**Figure 8:**
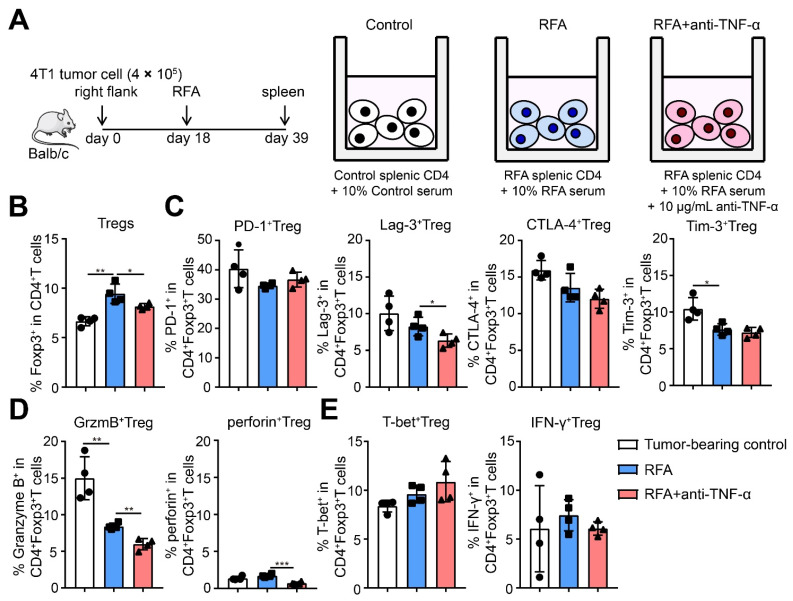
TNF-α neutralization after RFA treatment inhibited Treg function and suppressed tumor metastasis, as well as promoted the long-term survival of mice. (**A**) Schematic of experimental design. Splenic CD4^+^ T cells were separated from tumor-bearing control (39 days after inoculation) or RFA treated mice (21 days after treatment) and cultured in vitro for 24 h. The serum from tumor-bearing or RFA treated mice was added in corresponding group. An anti-TNF-α antibody was added in the concentration of 10 μg/mL. The proportion of Tregs (**B**), the expression of inhibitory checkpoints (**C**), the expression of suppressive cytokines (**D**), and the expression of T-bet, IFN-γ of Tregs (**E**) were detected by flow cytometry. *n* = 4 for each group. * *p* < 0.05, ** *p* < 0.01, *** *p* < 0.001.

Then, the proportion and suppressive signature of Tregs after cryo-thermal therapy with anti-TNF-α antibody in vitro were determined. The proportion of Tregs in splenic CD4^+^ T cells from RFA-treated mice was upregulated, but the proportion of Tregs was downregulated after RFA with TNF-α neutralization compared to RFA alone (Figure 8B). At the same time, the expression of Tim-3 and Granzyme B was downregulated on Tregs from RFA-treated mice (Figure 8C,D). After cryo-thermal therapy with TNF-α neutralization, the expression of Lag-3, Granzyme B, and perforin was decreased compared to RFA alone. However, there were no obvious changes in the expression of T-bet and IFN-γ after RFA with TNF-α neutralization (Figure 8E). All the in vivo and vitro results indicated that the higher level of TNF-α after RFA treatment increased the accumulation of Tregs and enhanced the suppressive function of Tregs.

## 3. Discussion

In this study, we revealed the effects of decreased serum TNF-α on the fragility and function of Tregs after cryo-thermal therapy in a s.q. 4T1 breast cancer model. Our results indicated that cryo-thermal therapy could inhibit tumor metastasis and improve long-term survival, induce the differentiation of CD4^+^ T cells toward a Th1 polarizing phenotype, downregulate the proportion and suppressive function of Tregs and drive the fragility of Tregs. Through TNF-α supplementation after cryo-thermal therapy and TNF-α neutralization after RFA treatment in vivo and in vitro, we further verified that the level of serum TNF-α was downregulated in the late phase after cryo-thermal therapy compared with conventional RFA treatment, which significantly played a crucial role in driving the fragility of Tregs and attenuating the suppressive function of Tregs. Conversely, the higher level of TNF-α in the late phase after RFA treatment increased the accumulation of Tregs and enhanced the suppressive function of Tregs.

Th1 cells expressing IFN-γ is the major subset that orchestrate the antitumor immune response by activating efficient antigen presentation and costimulatory molecule expression of APCs and the generation of cytolytic capacity of NK and CD8^+^ T cells [10]. In contrast, Tregs contribute to cancer progression by suppressing the antitumor immune response and are often associated with poor prognosis in tumor patients [12]. Moreover, Tregs induce lung metastasis in a s.q. 4T1 murine breast cancer model [28]. In our previous study, cryo-thermal therapy was proven to promote Th1 differentiation and reduce the proportion of Tregs in the B16F10 model [15]. In this study, we also found Th1-dominant differentiation of CD4^+^ T cells and downregulation of Tregs after cryo-thermal therapy in a s.q. 4T1 murine breast cancer model. Moreover, we also found that after cryo-thermal therapy, Tregs highly expressed functional markers of Th1 subsets (T-bet and IFN-γ), driving the fragility of Tregs. Fragile Tregs were represented by the upregulation of T-bet and IFN-γ but still maintained the expression of Foxp3 [23,24]. These Th1-like Tregs that lose their inhibitory function are the key element that may determine the response to immunotherapy [23]. Thus, cryo-thermal therapy drives the fragility of Tregs to enhance antitumor immunity, contributing to inhibiting tumor metastasis and improving long-term survival.

Sufficient IFN-γ expression can drive Treg fragility through the activation of the IFN-γ receptor and STAT1/STAT4 signaling [23,24]. Exogenous IFN-γ or inducing intratumoral production of IFN-γ increases IFN-γ and the expression of IFNγR on Tregs and limits the capacity of Tregs to suppress T cell proliferation [23]. However, whether other cytokines contribute to the fragility of Tregs has not been addressed. TNF-α has an important role in maintaining stability, promoting proliferation and improving the suppressive function of Tregs [29,30,31]. Moreover, TNF-α could increase the sensitivity of IL-2 signaling and facilitate the proliferation of Tregs [32]. TNF-α blockade leads to the loss of Foxp3 expression in Tregs [33], prevents Tim-3 expression on TILs and enhances the therapeutic effect of anti-PD-1 treatment [34]. However, whether TNF-α impacts the fragility of Tregs remains unclear. In this study, we confirmed that the level of serum TNF-α was significantly downregulated after cryo-thermal therapy compared to RFA treatment, which significantly played a major role in driving the fragility of Tregs and attenuating the suppressive function. Our previous studies showed that cryo-thermal therapy induced a high proportion of Th1 cells with high IFN-γ production, which drove the fragility of Tregs. Meanwhile, TNF-α is critical for maintaining the stability of Foxp3 expression on Tregs in Th1 polarizing culture conditions [31]. IFN-γ-rich and TNF-α-poor conditions after cryo-thermal therapy would be favorable for driving Treg fragility. However, an IFN-γ-poor and TNF-α-rich environment after RFA treatment enhances the suppressive function of Tregs. Nevertheless, this study suggested that the level of serum TNF-α in the late phase after therapy could be regarded as a biomarker of poor prognosis, and the combination of TNF-α blockade could improve the therapeutic effects of RFA in solid tumor treatments.

CD8^+^ T cells are consistently considered the major killing cells in tumors; however, increasing evidence shows that CD4^+^ T cells are the orchestrators of the antitumor immune response [35]. CD4^+^ T cells are capable of priming the immune response of CD8^+^ T cells and enhancing the function of innate immune cells [7]. In this study, we found that cryo-thermal therapy could promote Th1-dominant CD4^+^ T cell differentiation, but a higher level of IFN-γ expression in CD8^+^ T cells after RFA treatment was found compared to cryo-thermal therapy. In our previous study, we also found that the tumor-specific immune response was mainly induced by CD4^+^ T cells after cryo-thermal therapy in a B16F10 model [15,16]. Recent studies have shown that CD4^+^ T cells can clear tumors completely independently of CD8^+^ T cells and may even reduce the activation of CD8^+^ T cells in an IFN-γ-rich environment [36,37]. Thus, Th1-dominant CD4^+^ T cells with high expression of IFN-γ after cryo-thermal therapy reduced the reactivity of CD8^+^ T cells, while after RFA treatment, CD8^+^ T cells were the major cytotoxic T cells due to insufficiency of the Th1 immune response.

In this study, we found that the level of serum TNF-α in cryo-thermal treated mice was significantly increased at an early stage (3 days after cryo-thermal therapy). Acute TNF-α facilitated the recruitment of innate immune cells by promoting the release of chemokines, induced the production of inflammatory cytokines, and promoted the M1 polarization of macrophages and the maturation of DCs [38]. Therefore, the upregulation of TNF-α at an early stage after cryo-thermal therapy provided an acute inflammatory environment that aroused a strong and long-lasting antitumor immune response. However, which subset contributed to the change of TNF-α level at late stage after cryo-thermal therapy remains unknown. In this study, we found that the proportion of CD3^−^CD4^+^ cells was decreased after cryo-thermal therapy (Figure 2A). CD3^-^CD4^+^ is a heterogeneous group. CD4 has been reported to express in T cells (CD3^−^CD4^+^ T cells) [39], B cells [40], NKs [41], DCs [42], macrophages [43], monocytes [44], eosinophils [45], etc. Among these cells, CD4+ NKs, DCs, macrophages and monocytes have been reported to express TNF-α [41,42,43,44]. However, which subsets contributed to the change of blood TNF level after treatment would be discussed in the future.

In conclusion, we discovered that cryo-thermal therapy induced Th1-dominant differentiation and specifically drove Treg fragility by downregulating TNF-α level to achieve a long-term antitumor immune response. This study highlighted the benefits of cryo-thermal therapy for local tumor treatment, reprogramming the tumor suppressive environment, and eliciting a strong and durable systemic antitumor immunity, which will facilitate the development of combination strategies in immunotherapy.

## 4. Materials and Methods

### 4.1. Cell Culture and Animal Model

The female BALB/c mice were obtained from Shanghai Slaccas Experimental Animal Co., Ltd. (Shanghai, China). Mice were housed in isolated cages at a 12 h light/dark cycle environment, and were fed with sterile food and acidified water. All animal experiments were approved by the Animal Welfare Committee of Shanghai Jiao Tong University and experimental methods were performed in accordance with the guidelines of Shanghai Jiao Tong University Animal Care (approved by Shanghai Jiao Tong University Scientific Ethics Committee). To prepare the tumor-bearing mice, the 6–8 weeks mice were injected with approximately 4 × 10^5^ 4T1 cells subcutaneously into the right femoral region of each mouse. The 4T1 breast cancer cell line was provided by Shanghai First People’s Hospital, China. Cells were cultured in DMEM (Hyclone, Logan, UT, USA) supplemented with 10% FBS (GEMENI) and 100 U/mL penicillin, and 100 μg/mL streptomycin (Hyclone, USA). Cells were cultured at 37 °C in a 5% CO_2_ incubator. Eighteen days after inoculation, the diameter of tumor reached about 10 mm, and volume of tumor is about 0.2 cm^3^. Tumor volume was estimated using the following formula: V (mm^3^) = π/6 × L × W × H.

### 4.2. The Cryo-Thermal Therapy and RFA Procedures

The cryo-thermal therapy system was developed by our laboratory, and involves liquid nitrogen (LN_2_) for cooling and radiofrequency (RF) for heating, as well as a thermocouple for real-time feedback of the bottom tumor temperature. On day 18 after tumor inoculation, the diameter of the tumor reached approximately 10 mm, and the mice were anesthetized with 5% chloral hydrate (Sinopharm Chemical Reagent Co., Ltd., Shanghai, China). The tumors of mice in the cryo-thermal group were treated with fast LN_2_ freezing at −20 °C for 5 min followed by RF heating at 50 °C for 10 min. The tumors of mice in the conventional RFA group were treated with RF heating at 65 °C for 15 min. All heating and freezing processes were controlled within 1 min.

### 4.3. H&E Staining and Immunohistochemistry

On day 21 after treatments, the lungs of mice from each group were harvested and fixed in 4% formaldehyde. Then, the specimen was paraffin embedded and sliced into 5 μm sections. The sections were stained with hematoxylin and eosin, and then pathological changes were observed under a light microscope. For immunochemistry staining, sections were dewaxed in xylene and hydrated through graded alcohols. Antigen repair was performed by boiling the slides in a microwave for 20 min in sodium citrate buffer, and then endogenous catalase was blocked with 3% hydrogen peroxide methanol solution. Next, the sections were treated with 0.3% Triton-X 100 for 10 min followed by 5% bovine serum albumin blockade for 1 h at 37 °C. Sections were incubated with TNF-α antibodies (diluted at 1:100, BD, Franklin Lakes, NJ, USA) at 4 °C overnight. An anti-mouse IgM SABC kit (Boster, Wuhan, China) was used instead of other secondary antibodies. The sections were then stained with 3,3′-diaminobenzidine (Beyotime, Shanghai, China), and the nuclei were stained with hematoxylin.

### 4.4. Analysis of TNF-α in Mice Serum

The sera of each group were collected at 1, 3, 7, 14, and 21 days after treatment. One microliter of serum from each group was separated on 12% gradient Tris-glycine precast gels and transferred to PVDF membranes. The blot was probed with anti-TNF-α (Biolegend, San Diego, CA, USA). Albumin was used as the internal reference and was stained with Ponceau S. Each Western blot result shown is representative of three separate experiments.

The level of TNF-α in mouse serum from each group on day 21 after treatment was analyzed using a mouse TNF-α ELISA kit (Boster, Wuhan, China). A standard curve was established according to the manufacturer’s instructions.

### 4.5. Flow Cytometry Analysis

The spleens were collected after therapy and separated to obtain a single-cell suspension of splenocytes through a GentleMACS dissociator (Miltenyi Biotec, Bergisch Gladbach, Germany). Erythrocyte-lysing reagent (containing 0.15 M NH_4_Cl, 1.0 M KHCO_3_, and 0.1 mM Na_2_EDTA) was used to remove red blood cells. The cells were dispersed using 70 μm mesh screens.

For the detection of transcription factors, cells were stained with Zombie Dye to exclude dead cells, followed by cell-specific surface marker staining for 20 min. Next, the cells were stained according to the manufacturer’s protocol using the True-Nuclear Transcription Factor Buffer Set (Biolegend, USA). The cells were then incubated with antibodies against the transcription factors for 45 min. For intercellular staining, cells were stimulated with Cell Activation Cocktail (with Brefeldin A, 20 µg/mL, Biolegend, USA) for 4 h. After stimulation, the Zombie Dye staining and cell-specific surface marker staining was conducted in the same manner as that of transcription factor detection. Next, the cells were fixed with fixation buffer (Biolegend, USA) and permeabilized with intracellular staining permeabilization wash buffer (Biolegend, USA) according to the manufacturer’s instructions. The cells were then incubated with antibodies binding specific intercellular markers for 20 min. A BD FACS Aria II cytometer (BD Biosciences) was used for data collection, and the data were analyzed using FlowJo software. Fluorochrome-conjugated monoclonal antibodies: CD3-Percp/cy5.5 (clone 145-2C11), CD4-APC/cy7 (clone RM4-5), CD8-Pacific blue (clone 53-6.7), PD-1-PE/cy7 (clone 29F.1A12), CD25-PE/cy7 (clone 3C7), Lag-3-Percp/cy5.5 (clone C9B7W), Tim-3-BV421 (clone RMT2-23), IL-4-BV421 (clone 11B11), IL-17-APC (clone TC11-18H10.1), Perforin-PE (clone S16001A), Granzyme-B-AF647 (clone GB11), Bcl-6-BV421 (clone K112-91), Foxp3-PE (clone MF-14), T-bet-PE/cf594 (clone 4B10), GATA3-AF488 (clone 16E10A23) (all from Biolegend, USA). IFN-γ-PE/cf594 (clone XMG1.2), Thpok-AF647 (clone T43-94) (from BD Biosciences, USA). CTLA-4-PE (clone UC10-4B9) (from eBioscience, San Diego, CA, USA).

### 4.6. Isolation of CD4^+^, CD8^+^ T Cells and TREGS

For isolation of CD4^+^ and CD8^+^ T cells, spleens from the treated mice and 4T1 tumor-bearing control mice were harvested on day 21 after treatment, and single cell suspensions were prepared using a GentleMACS dissociator (Miltenyi Biotec). CD4^+^ and CD8^+^ T cells were isolated by an Easysep CD4^+^ T cell negative selection kit and an Easysep CD8^+^ T cell negative selection kit (StemCell Technologies, Vancouver, BC, Canada), respectively. CD4^+^ and CD8^+^ T cells with a purity of >90% were used for experiments. For isolation of Tregs, the isolated CD4^+^ T cells were then incubated with CD25-PE (clone 3C7, Biolegend, USA) and isolated with a PE positive selection kit (StemCell Technologies, Canada). The isolated cells were then verified by flow cytometry, and more than 85% of cells expressed Foxp3.

### 4.7. Proliferation Assay

Carboxyfluorescein succinimidyl ester (CFSE) was used to measure the cell proliferation activity of tumor-bearing CD4^+^ and CD8^+^ T cells. After cell isolation, 1 × 10^7^ CD4^+^ or CD8^+^ T cells were incubated with CFSE at a final concentration of 5 μM for 30 min, washed twice, and then cultured in RPMI 1640 (Thermo Fisher, Waltham, MA, USA) with anti-CD3 (1 ng/mL, Biolegend, USA) for 72 h. CFSE dilution was measured by flow cytometry. As the CFSE signal is diluted with each cell division, cells exhibiting a high CFSE fluorescence intensity were considered not to be proliferative, and a low CFSE fluorescence intensity is considered to proliferate.

### 4.8. In Vitro Tregs Suppressive Function Assay

Splenic CD8^+^ T cells were isolated from age-paired naive mice, and CD8^+^ T cells were then stained with CFSE. Splenic Tregs were isolated from tumor-bearing control, RFA- or cryo-thermal treated mice on day 21 after treatment. Tregs from different mouse groups and CD8^+^ T cells were cocultured at different E/T ratios with anti-CD3 (1 ng/mL) for 72 h. The proliferation of CD8^+^ T cells was analyzed by flow cytometry. Tregs from different mouse groups were cocultured at a ratio of 4:1 with anti-CD3 (1 ng/mL) for 24h.

### 4.9. In Vivo TNF-α Blockade and TNF-α Recombinant Protein Injection

The 4T1 tumor-bearing mice were generated as described above and randomly divided into three groups: tumor-bearing control, RFA, and cryo-thermal therapy. On days 10 and 14 after treatment, the mice in the cryo-thermal therapy and RFA treatment groups were separated into three groups and treated with PBS, anti-TNF-α antibody (150 μg in 100 μL PBS) or TNF-α recombinant protein (200 ng in 100 μL PBS) by intraperitoneal injection.

### 4.10. Statistical Analysis

The student’s *t*-test was used for statistical comparisons using Graph Pad Prism 7. Figures denoted statistical significance of * *p* < 0.05, ** *p* < 0.01, *** *p* < 0.001, **** *p* < 0.0001. *p*-values < 0.05 was considered to be statistically significant.

## Data Availability

Data are contained within the article or Supplementary Material or are available from the authors upon reasonable request.

## Supplementary Materials

The following are available online at https://www.mdpi.com/article/10.3390/ijms22189951/s1, Figure S1: Cryo-thermal mediated antitumor immune response was not depended on CD8+ T cells, Figure S2: Cryo-thermal therapy drove the fragile phenotype of Tregs and down-regulated its suppressive function, Figure S3: In vivo TNF-α supplement after cryo-thermal therapy damaged Tregs fragility and promoted tumor metastasis of mice, Figure S4: In vivo TNF-α supplement after cryo-thermal therapy damaged Tregs fragility and promoted tumor metastasis of mice, Figure S5: TNF-α neutralization after RFA treatment inhibited Treg function and suppressed tumor metastasis as well as promoted the long-term survival of mice.
