# Supplementary material for: Downregulated TNF-α Levels after Cryo-Thermal Therapy Drive Tregs Fragility to Promote Long-Term Antitumor Immunity
Source: Int J Mol Sci. 2021 Sep 15;22(18):9951. doi: 10.3390/ijms22189951 (PMC8468796; doi:10.3390/ijms22189951)
Supplement: Supplementary file 1 [file ijms-22-09951-s001.zip › ijms-1364452-supplementary.pdf]

Supplementary materials

Downregulated TNF-α levels after cryo-thermal therapy drive Tregs fragility to promote long-term antitumor immunity

Yue Lou [1], Junjun Wang [1], Peng Peng [1], Shicheng Wang [1], Ping Liu [1,*] and Lisa X. Xu [1,*]

[1] School of Biomedical Engineering and Med-X Research Institute,Shanghai Jiao Tong University, Shanghai, China

* Correspondence: Lisa X. Xu: lisaxu@sjtu.edu.cn; Ping Liu: pingliu@sjtu.edu.cn

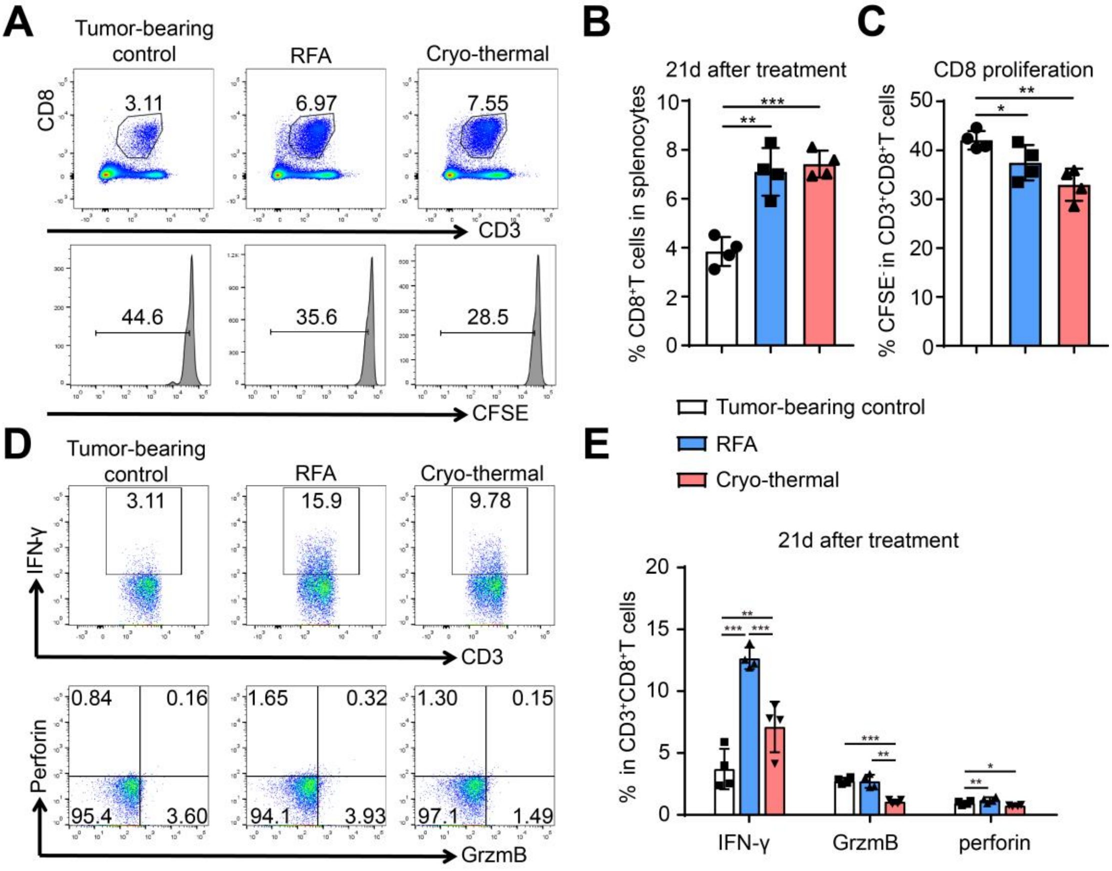

Figure S1. Cryo-thermal mediated antitumor immune response was not depended on CD8$^+$ T cells. (A-C) The percentages of CD8$^+$ T cells in splenic lymphocytes (B) and the proliferation (C) of CD8$^+$ T cells were detected by flow cytometry on day 21 after cryo-thermal therapy or RFA treatment. For CD8$^+$ T cells proliferation assay. Splenic CD8$^+$ T cells were isolated from tumor-bearing control (39 days after inoculation), RFA or cryo-thermal (21 days after treatment) treated mice by using microbeads. The CD8$^+$ T cells were labeled with CFSE and then cultured with αCD3 (1 ng/mL) for 24h. CD8$^+$ T cell proliferation (CFSE-) were detected by flow cytometry. (D) The expression of IFN-γ, Granzyme B and perforin in splenic CD8$^+$ T cells were detected by flow cytometry. *$P < 0.05$, **$P < 0.01$, ***$P < 0.001$. n = 4 for each group.

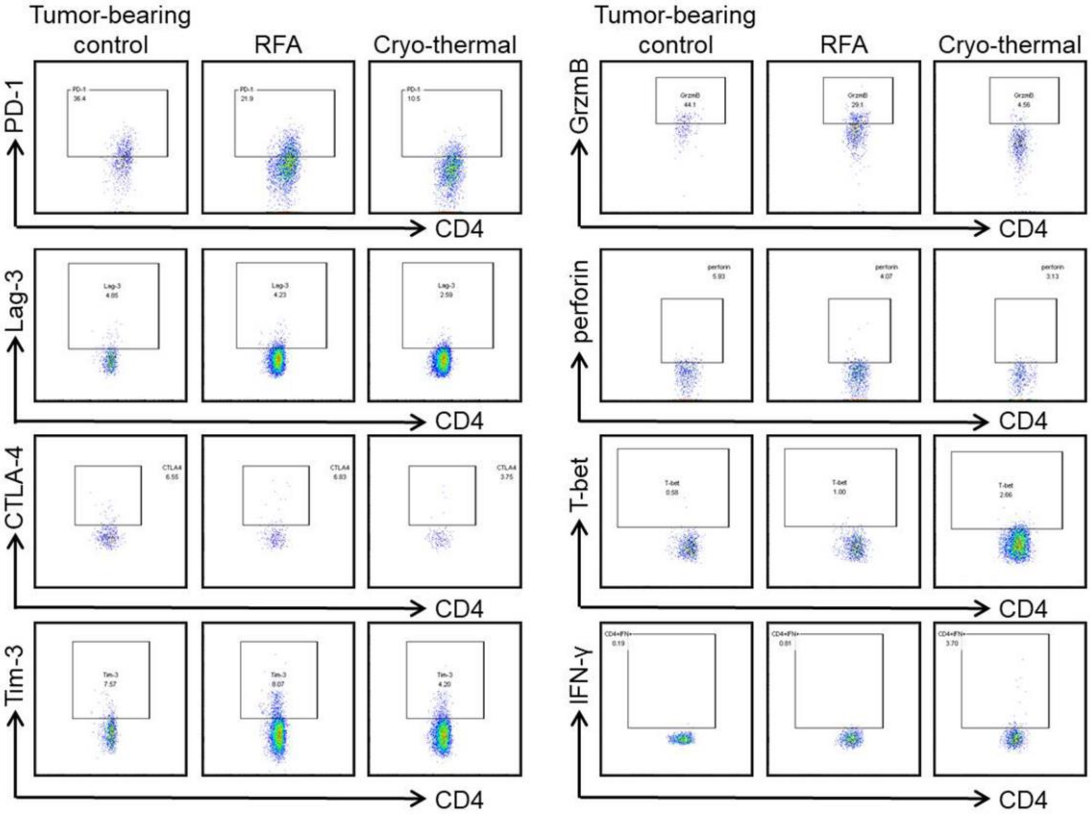

Figure S2. Cryo-thermal therapy drove the fragile phenotype of Tregs and down-regulated its suppressive function. Flow cytometry gating strategy for determination of PD-1, Lag-3, CTLA-4, Tim-3, Granzyme B, Perforin, T-bet and IFN-γ in Tregs. (Representative flow chart of Fig. 3).

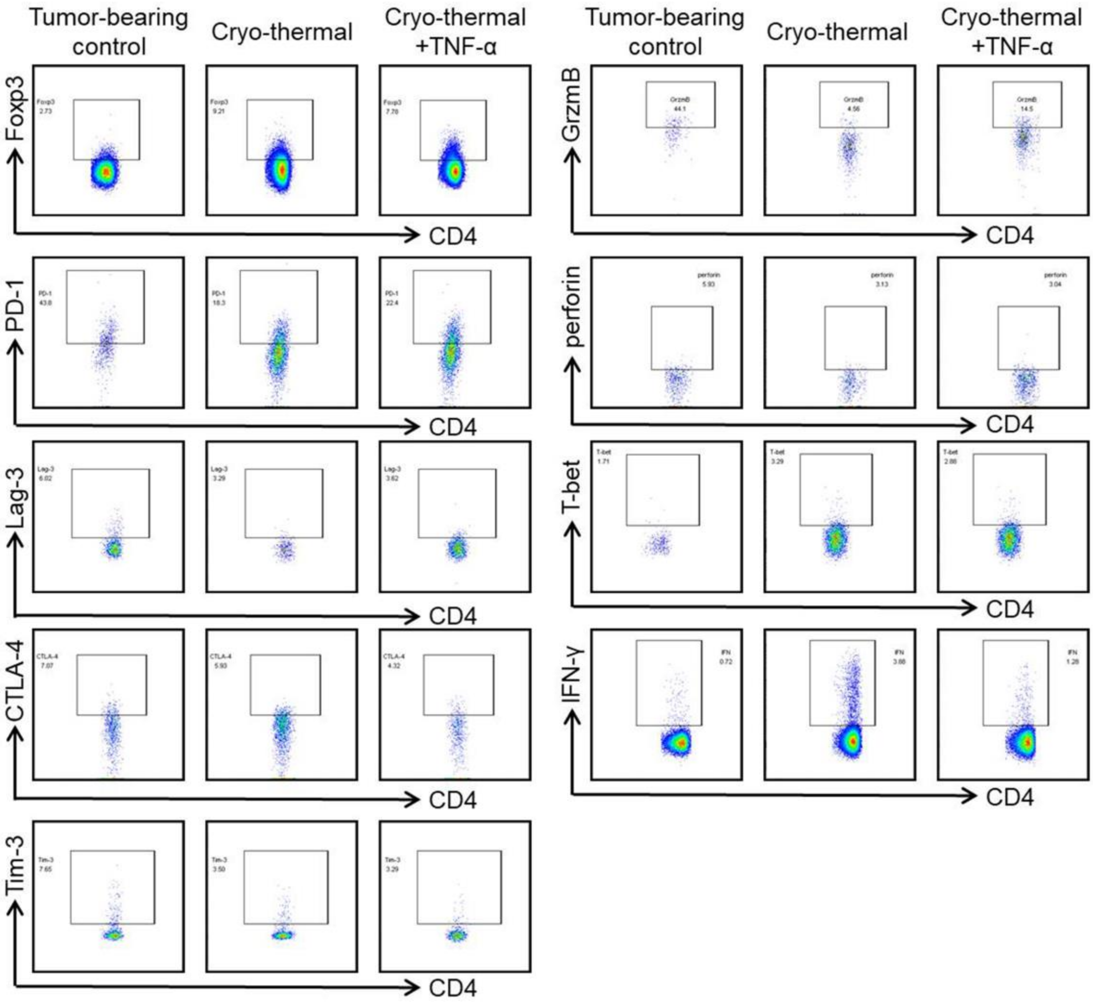

Figure S3. *In vivo* TNF-α supplement after cryo-thermal therapy damaged Tregs fragility and promoted tumor metastasis of mice. Flow cytometry gating strategy for determination of Tregs, and the expression of PD-1, Lag-3, CTLA-4, Tim-3, Granzyme B, Perforin, T-bet and IFN-γ in Tregs. (Representative flow chart of Fig. 5).

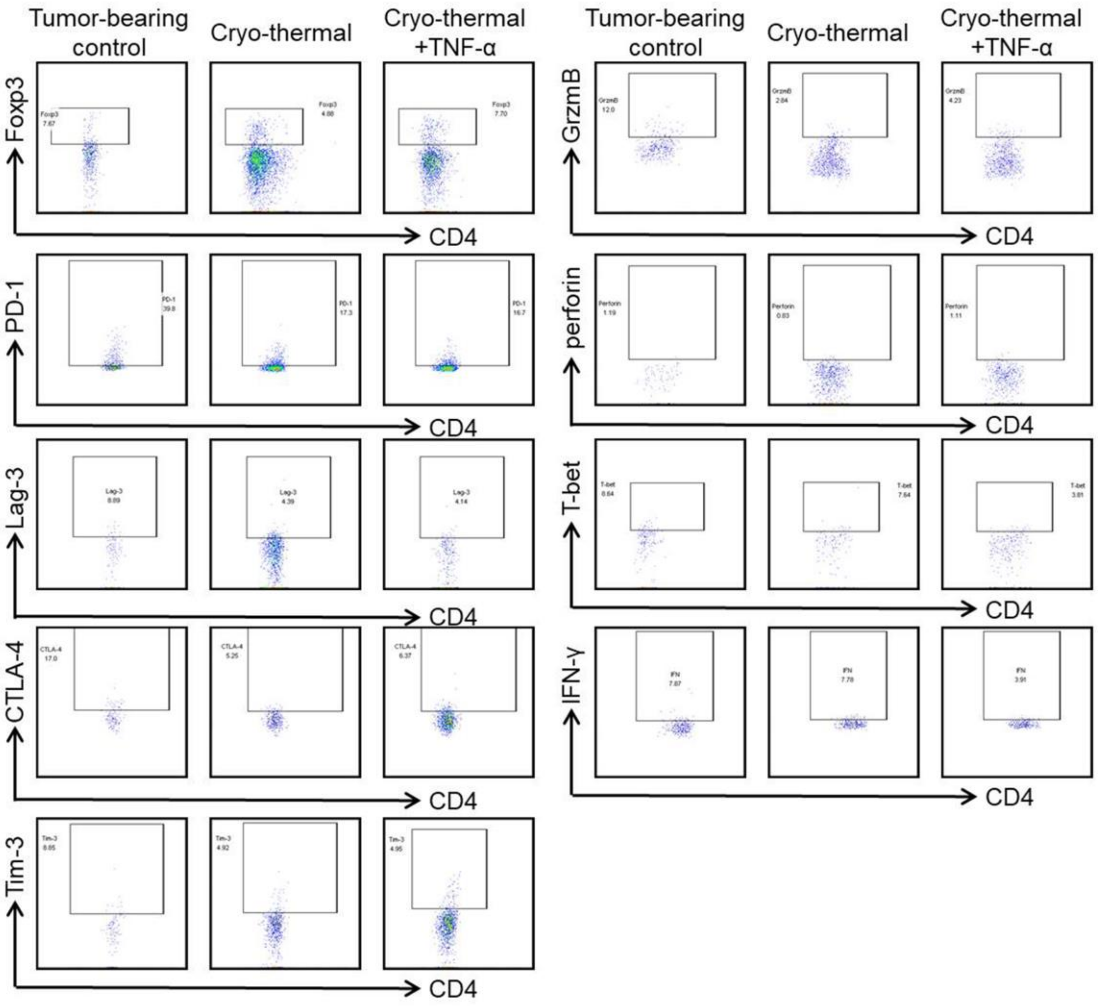

Figure S4. *In vivo* TNF-α supplement after cryo-thermal therapy damaged Tregs fragility and promoted tumor metastasis of mice. Flow cytometry gating strategy for determination of Tregs, and the expression of PD-1, Lag-3, CTLA-4, Tim-3, Granzyme B, Perforin, T-bet and IFN-γ in Tregs. (Representative flow chart of Fig. 6).

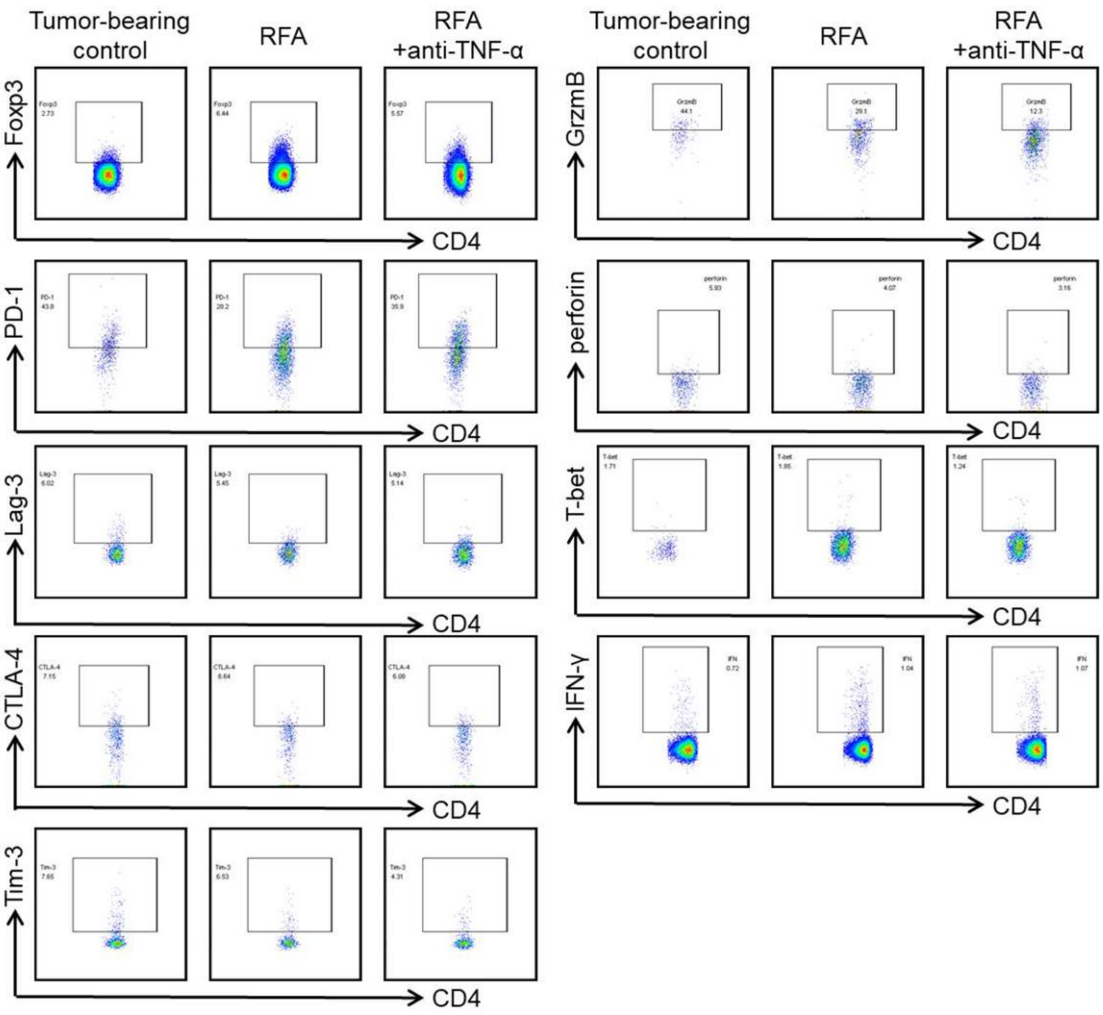

Figure S5. TNF-α neutralization after RFA treatment inhibited Treg function and suppressed tumor metastasis as well as promoted the long-term survival of mice. Flow cytometry gating strategy for determination of Tregs, and the expression of PD-1, Lag-3, CTLA-4, Tim-3, Granzyme B, Perforin, T-bet and IFN-γ in Tregs. (Representative flow chart of Fig. 7).

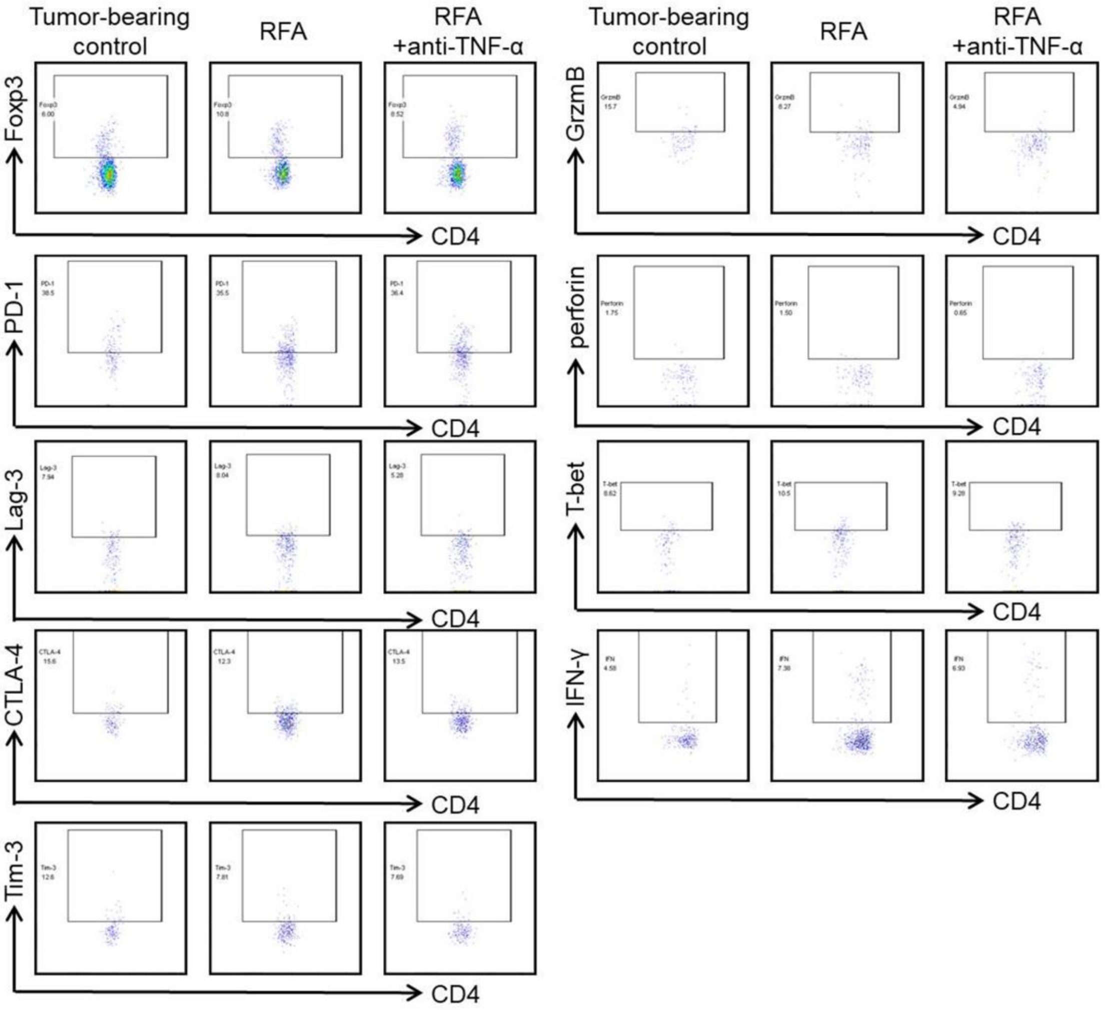

Figure S6. TNF-α neutralization after RFA treatment inhibited Treg function and suppressed tumor metastasis as well as promoted the long-term survival of mice. Flow cytometry gating strategy for determination of Tregs, and the expression of PD-1, Lag-3, CTLA-4, Tim-3, Granzyme B, Perforin, T-bet and IFN-γ in Tregs. (Representative flow chart of Fig. 8).
